# Development and Validation of a Predictive Model for Severe COVID-19: A Case-Control Study in China

**DOI:** 10.3389/fmed.2021.663145

**Published:** 2021-05-25

**Authors:** Zirui Meng, Minjin Wang, Zhenzhen Zhao, Yongzhao Zhou, Ying Wu, Shuo Guo, Mengjiao Li, Yanbing Zhou, Shuyu Yang, Weimin Li, Binwu Ying

**Affiliations:** ^1^Department of Laboratory Medicine, West China Hospital, Sichuan University, Chengdu, China; ^2^Department of Respiratory and Critical Care Medicine, West China Hospital, Sichuan University, Chengdu, China

**Keywords:** COVID-19, severe COVID-19, predictive model, laboratory findings, cytokines, online predictive calculator

## Abstract

**Background:** Predicting the risk of progression to severe coronavirus disease 2019 (COVID-19) could facilitate personalized diagnosis and treatment options, thus optimizing the use of medical resources.

**Methods:** In this prospective study, 206 patients with COVID-19 were enrolled from regional medical institutions between December 20, 2019, and April 10, 2020. We collated a range of data to derive and validate a predictive model for COVID-19 progression, including demographics, clinical characteristics, laboratory findings, and cytokine levels. Variation analysis, along with the least absolute shrinkage and selection operator (LASSO) and Boruta algorithms, was used for modeling. The performance of the derived models was evaluated by specificity, sensitivity, area under the receiver operating characteristic (ROC) curve (AUC), Akaike information criterion (AIC), calibration plots, decision curve analysis (DCA), and Hosmer–Lemeshow test.

**Results:** We used the LASSO algorithm and logistic regression to develop a model that can accurately predict the risk of progression to severe COVID-19. The model incorporated alanine aminotransferase (ALT), interleukin (IL)-6, expectoration, fatigue, lymphocyte ratio (LYMR), aspartate transaminase (AST), and creatinine (CREA). The model yielded a satisfactory predictive performance with an AUC of 0.9104 and 0.8792 in the derivation and validation cohorts, respectively. The final model was then used to create a nomogram that was packaged into an open-source and predictive calculator for clinical use. The model is freely available online at https://severeconid-19predction.shinyapps.io/SHINY/.

**Conclusion:** In this study, we developed an open-source and free predictive calculator for COVID-19 progression based on ALT, IL-6, expectoration, fatigue, LYMR, AST, and CREA. The validated model can effectively predict progression to severe COVID-19, thus providing an efficient option for early and personalized management and the allocation of appropriate medical resources.

## Introduction

The current outbreak of coronavirus disease 2019 (COVID-19) has spread rapidly and widely across the world, causing panic and major public health challenges in the international community (1). COVID-19 presents a wide clinical manifestation, including asymptomatic infection, mild upper respiratory tract illness, and severe viral pneumonia, with respiratory failure. Only a small proportion of the total number of cases progress to a severe condition (~15–20%); however, ~40% of patients with severe disease die (2–5). Although some research has shown that initial therapy with remdesivir or non-invasive positive pressure ventilation (NIPPV) is very efficient for severe cases, there is currently a lack of accepted recommendations for severe patients with regard to individualized treatment (6–8). Therefore, the rapid deterioration of patients with severe COVID-19 deserves special attention. There is an urgent need to develop options for the personalized diagnosis and treatment of such patients, particularly with regard to protecting the relative shortage of medical resources.

Fever, cough, and fatigue are commonly present in patients with mild COVID-19 (9, 10). As the disease progresses further, patients may also experience respiratory failure, acute respiratory distress syndrome, heart failure, metabolic acidosis, and septic shock (11). Besides the well-defined clinical characteristics of COVID-19, previous studies have shown that abnormal laboratory findings and cytokine levels are often associated with disease progression, including coagulation-related markers such as D-dimer and fibrinogen (FIB), neutrophil count, lymphocyte count, and high-sensitivity C-reactive protein (HsCRP) (5, 12–15). In addition, research has identified that a cytokine storm could be the primary driver of severe progression in COVID-19 patients (16, 17). However, the application of these independent indicators is limited by many factors, including insufficient information, individual differences, the experience of the attending physician, and the complexity of disease. Thus, there is an urgent need for advanced multivariable prediction models (18, 19). Although several studies have attempted to develop prediction models, most of the existing models were developed in a single center and based on retrospective data; in some cases, only partial datasets were used, and there was a clear lack of validation. These factors may lead to the omission of key variables and the risk of over-fitting, thus limiting the clinical application of such models. Therefore, there is a critical need to develop more effective prediction models (14, 15, 20, 21).

Here, we prospectively and consecutively enrolled a cohort of COVID-19 patients with a complete set of demographic data, clinical characteristics, laboratory findings, and cytokine information, and we then constructed a multiparameter prediction model for the early identification of severe COVID-19. Our model could help to monitor and guide precision medicine.

## Methods

### Participants

**Figure 1:**
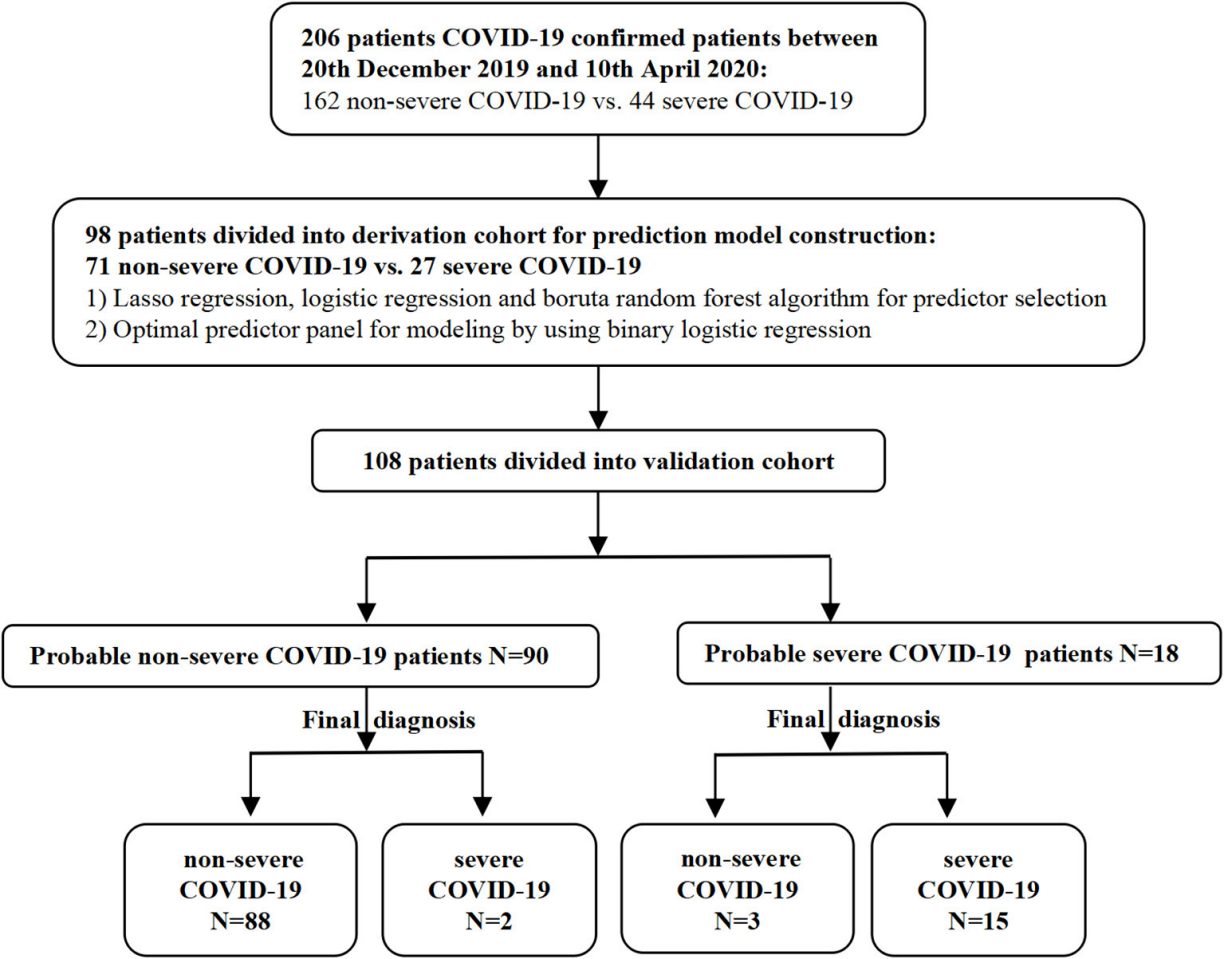
Study flowchart.

COVID-19 patients were prospectively and consecutively enrolled from regional medical institutions by the West China Medical Center between December 20, 2019, and April 10, 2020. The patients were divided into severe and non-severe groups according to the China National Health Commission Guidelines for Diagnosis and Treatment of COVID-19 infection (Versions 5 and 7). Serum samples were collected from patients within 3 days of infection confirmation and stored at −80°C for the subsequent detection of cytokine levels. Demographic data, clinical characteristics, and laboratory findings were acquired from electronic medical records (Figure 1). Two independent researchers reviewed the data collection forms.

### Diagnostic and Severity Classification Criteria

Patients with pneumonia, typical findings on computed tomography (CT) chest scan, and positive severe acute respiratory syndrome coronavirus 2 (SARS-CoV-2) nucleic acid results, as determined by real-time fluorescent reverse transcription-polymerase chain reaction assessment from bronchoalveolar lavage (BAL) or sputum, were considered as COVID-19 “cases” according to the diagnosis and treatment guidelines released by the China Health and Medical Commission (22). Patients with at least one of the following symptoms during hospitalization were allocated into the severe group: (1) respiratory distress, respiratory rate ≥30 times/min; (2) oxygen saturation ≤93% at rest; and (3) oxygen partial pressure (PaO_2_)/oxygen concentration (FiO_2_) in arterial blood ≤300 mmHg. All patients were discharged or had died by the time the model was developed.

### The Detection of Cytokines

Circulating levels of interferon (IFN)-α2, IFN-β, IFN-γ, tumor necrosis factor (TNF)-α, interleukin (IL)-1α, IL-1β, IL-2, IL-4, IL-6, IL-8, IL-10, IL-17A, IL-17E, IL-17F, IL-22, and IL-33 in serum samples were measured by a multiplexed flow cytometric assay using Human Cytokine Kits on a Luminex® system (MAGPIX® with xPONENT) according to the manufacturer's instructions (MILLIPLEX® Analyst 5.1). All samples were measured in duplicate. Based on standard curves, we calculated the coefficient of variation (CV); this did not exceed 20%.

### Construction of the Predictive Model and Internal Validation

Patients from the Chengdu region were divided into a derivation cohort, including a training set for modeling and a testing set for internal validation. Stepwise selection was based on *p*-values; least absolute shrinkage and selection operator (LASSO) and the Boruta algorithm were used to select variables (23, 24). Stepwise selection, as based on *p*-values, is a classic regression-based method. A variable's value with a *p* < 0.05 was regarded as significant and was retained. This practice generally achieves a better performance in smaller datasets and has been extensively used in previous research. LASSO regression can compress the coefficients of the features *via* penalty function to obtain optimal constraint models; this practice has been used effectively to avoid over-fitting and co-linearity in classical analysis methods based on significance differences and also enhances the ability of a model to be generalized. Boruta algorithm is a wrapper algorithm that uses random forest classification. This practice can iteratively remove features that prove to be less relevant than random probes and thus aims to retain relevant variables for the function of a response variable. In addition, these two algorithms are particularly suitable for a dataset with a small sample size but with a large number of variables. By using these three different variable selection methods, we were able to select three candidate predictor panels to construct different binary logistic regression models, which were then verified internally by 10-fold cross-validation. The optimal model was then selected by comparing the area under the curve (AUC) and the Akaike information criterion (AIC) in order to generate a nomogram that could be encapsulated as an open-source online predictive calculator.

### Independent Validation

The independent validation cohort consisted of patients from outside Chengdu; this was used for external verification to predict the generalization ability of the model by comparing the predicted results with a set of follow-up results to calculate several metrics: sensitivity, specificity, positive predictive value (PPV), and negative predictive value (NPV). AUCs and decision curve analysis (DCA) were used to comprehensively evaluate the model's discrimination and net clinical benefits (25).

### Statistical Analysis

Continuous variables and categorical variables are presented as the median (upper and lower quartiles) and as a frequency, respectively. The chi-squared test for categorical variables and the Student *t*-test or Mann–Whitney *U*-test for continuous variables were used to test the data between groups. Pearson correlation was used to determine the linear correlation between two variables. The diagnostic performance of equations was then displayed by AIC and receiver operating characteristic (ROC) curve and quantified by AUCs. An open-source online predictive calculator was then created using the Shiny tool in the R environment (version 1.2.0). All statistical analyses were completed using R 3.5.0 version. All statistical tests were two-tailed, and *p* ≤ 0.05 was considered to indicate statistical significance.

### Standard Protocol Approvals, Registrations, and Patient Consent

The protocol for this study was approved by the West China Hospital, Sichuan University Medical Ethics Committee (reference no. 193, 2020), and conformed to the principles of the Declaration of Helsinki. Written informed consent was obtained from all participants.

## Results

### Epidemiological Characteristics

We recruited 206 patients with a confirmed diagnosis of COVID-19; of these, 44 patients progressed to severe COVID-19, and 162 patients were classified as having non-severe COVID-19. Patients in the severe group were significantly older (50 *vs*. 46, *p* = 0.005) and had a significantly higher frequency of underlying diseases (diabetes and hypertension) than the non-severe group (*p* < 0.001 and *p* = 0.013, respectively). There were no differences between the two groups in terms of gender (male: 54.940 *vs*. 56.810%, *p* = 0.400). With regard to epidemiological exposure, most of the patients (79.000%) in the severe group had been overseas or had visited Wuhan or surrounding regions within 14 days of disease onset; patients who had been overseas accounted for 50% of the patients with non-severe COVID-19. As of April 28, 2020, the time for the reversal of a negative nucleic acid test result in the non-severe and severe groups was 11 and 18 days (median) except for three patients who died from multiple organ failure (MOF).

### Differences in Characteristics and Correlation Analysis

Demographic data, clinical characteristics, laboratory findings, and cytokine levels are shown in Table 1 and Supplementary Figures 1, 2. Several cytokines were significantly elevated in the severe COVID-19 group (*p* ≤ 0.010). The predictive value of each single cytokine, and a combined panel of cytokines, were evaluated by ROC curve analysis and quantified by AUC (Supplementary Figure 3). Results showed that the AUCs were 0.830, 0.796, 0.729, 0.707, 0.694, 0.667, 0.656, and 0.653 for single IL-10, IL-6, IL-1α, IL-1β, IL-17A, IL-4, TNF-α, and IL-2 and that the binary logistic model had a similar AUC (0.796–0.848). These data indicated that IL-10 and IL-6 may represent potential biomarkers for patients with severe COVID-19. We found significant differences between the severe and non-severe COVID-19 group with regard to a range of clinical characteristics, including respiratory rate, cough, expectoration, dyspnea, asthma, and debilitation. Significant differences were also identified in several laboratory findings; lymphocyte ratio (LYMR), eosinophil ratio (EOSR), monocyte ratio (MONOR), total bilirubin (TBIL), total protein (TP), albumin (ALB), Ca, and URIC were all significantly lower in the severe COVID-19 group, while neutrophil ratio (NEUTR), FIB, aspartate transaminase (AST), glucose (GlU), and HsCRP were all significantly higher. However, the AUCs for these indicators when used to predict severe COVID-19 were all <0.690. Simple logistic analysis was not suited for the severe COVID-19 group, owing to the feature selection of such a large number of indicators.

**Table 1:** Patients' characteristics in training set.

	**Non-severe COVID-19**	**Severe COVID-19**	***P*-value**		**Non-severe COVID-19**	**Severe COVID-19**	***P*-value**
	**71 patients**	**27 patients**			**71 patients**	**27 patients**	
Demographics				Cytokines			
Age	46 (33, 51)	50 (42, 65)	0.005^*^	IFN-β	8.87 (8.08, 8.87)	8.87 (8.08, 8.87)	0.403
Sex	53.50%	63.00%	0.4	IFN-γ	10.58 (1.71, 24.88)	17.07 (3.67, 54.26)	0.161
Diabetes	4.20%	29.60%	<0.001^*^	Laboratory findings			
Hypertension	16.90%	40.70%	0.013^*^	WBC	5.41 (4.40, 7.15)	6.31 (4.40, 7.67)	0.148
Clinical features				HB	137.00 (126.00, 156.00)	134.00 (125.00, 151.00)	0.239
Temperature	36.7 (36.5, 37.4)	37.2 (36.7, 37.7)	0.115	PLT	180.00 (141.00, 244.00)	146.00 (120.00, 223.00)	0.131
Heart rate	88 (78, 97)	90 (84, 105)	0.196	LYMR	23.80 (18.80, 30.90)	15.20 (5.10, 23.40)	<0.001^*^
Respiratory rate	20 (20, 21)	20 (20, 22)	0.008^*^	NEUTR	65.80 (57.60, 71.00)	77.00 (67.20, 87.60)	<0.001^*^
SBP	130 (120, 140)	139 (123, 150)	0.183	EOSR	0.20 (0.09, 0.90)	0.00 (0.00, 0.20)	0.003^*^
DBP	82 (77, 89)	84 (76, 94)	0.619	BASOR	0.20 (0.10, 0.30)	0.20 (0.10, 0.30)	0.977
SBP-DBP	48 (42, 58)	50 (44, 63)	0.202	MONOR	8.30 (6.80, 10.80)	7.00 (4.20, 9.10)	0.028^*^
Fever	60.60%	74.10%	0.212	HCT	39.80 (35.60, 45.40)	36.60 (29.50, 44.10)	0.215
Cough	45.10%	81.50%	0.001^*^	D-dimer	1.90 (0.32, 62.44)	5.46 (0.83, 31.00)	0.75
Dry cough	25.40%	25.90%	0.954	FIB	3.59 (2.84, 4.32)	4.03 (3.20, 4.86)	0.045^*^
Expectoration	16.90%	51.90%	<0.001^*^	APTT	31.82 (27.20, 37.80)	32.70 (31.20, 35.20)	0.578
Dyspnea	2.80%	25.90%	<0.001^*^	PT	12.40 (11.70, 13.40)	12.70 (11.90, 13.30)	0.559
Asthma	4.20%	18.50%	0.021^*^	INR	1.02 (0.97, 1.10)	1.05 (1.00, 1.15)	0.379
Chest distress	7.00%	11.10%	0.511	TBIL	10.70 (7.11, 14.90)	6.78 (4.00, 13.35)	0.043^*^
Nasal obstruction	2.80%	0.00%	*P* = 0.378	DBIL	3.40 (2.20, 4.60)	3.90 (2.64, 6.40)	0.272
Nasal discharge	5.60%	3.70%	*P* = 0.698	IBIL	6.60 (4.50, 11.70)	5.47 (2.70, 9.33)	0.158
Earache	0.00%	3.70%	0.103	ALT	29.50 (18.00, 48.01)	41.17 (21.00, 74.14)	0.147
Sore throat	11.30%	18.50%	0.344	AST	26.00 (19.60, 35.70)	37.00 (23.70, 70.62)	0.022^*^
Headache	16.90%	7.40%	0.23	TP	75.00 (70.70, 79.00)	68.41 (63.20, 77.30)	0.014^*^
Myalgia	8.50%	11.10%	0.684	ALB	44.30 (41.00, 46.67)	39.60 (34.90, 44.27)	0.001^*^
Arthralgia	1.40%	3.70%	0.473	GLB	30.20 (27.06, 33.05)	29.31 (23.80, 32.75)	0.057
Chest wall invagination	0.00%	0.00%	—	TG	1.66 (0.94, 2.28)	1.44 (0.84, 2.37)	0.507
Fatigue	4.20%	25.90%	0.002^*^	CHOL	4.20 (3.42, 4.80)	3.85 (2.87, 4.58)	0.088
Diarrhea	11.30%	14.80%	0.632	HDL-C	1.20 (1.03, 1.39)	1.04 (0.92, 1.43)	0.231
Cytokines				LDL-C	2.30 (1.84, 2.84)	1.87 (1.33, 2.60)	0.054
IL-1α	2.91 (1.47, 7.80)	7.05 (2.91, 11.53)	0.025^*^	CK	93.00 (56.80, 216.00)	217.76 (64.00, 314.30)	0.053
IL-1β	1.60 (0.66, 4.86)	3.24 (1.37, 7.80)	0.06	CK-MB	11.83 (5.32, 14.73)	12.34 (3.76, 17.00)	0.688
IL-2	0.21 (0.10, 0.45)	0.36 (0.14, 0.61)	0.343	Glu	5.63 (5.07, 6.97)	6.80 (5.44, 9.15)	0.044^*^
IL-4	1.94 (0.48, 3.91)	1.73 (0.78, 6.47)	0.181	Na	140.10 (137.10, 143.00)	138.00 (133.14, 141.80)	0.097
IL-6	1.19 (0.66, 3.54)	5.03 (2.12, 18.61)	<0.001^*^	K	3.97 (3.60, 4.30)	3.94 (3.40, 4.33)	0.519
IL-8	3.72 (2.18, 6.98)	5.56 (3.82, 9.44)	0.074	Ca	2.27 (2.16, 2.39)	2.09 (1.96, 2.19)	<0.001^*^
IL-10	1.13 (0.34, 2.47)	3.76 (0.92, 14.15)	0.001^*^	Mg	0.87 (0.82, 0.99)	0.85 (0.79, 1.02)	0.741
IL-17A	0.97 (0.52, 4.12)	1.45 (0.80, 4.37)	0.083	Urea	3.70 (2.90, 5.30)	4.31 (3.30, 7.20)	0.076
IL-17E	28.56 (17.37, 90.70)	24.36 (9.85, 80.88)	0.203	CREA	71.60 (56.00, 83.30)	69.40 (52.00, 192.90)	0.735
IL-17F	7.66 (5.75, 13.53)	7.66 (6.22, 10.75)	0.817	URIC	348.00 (258.00, 427.00)	276.00 (183.00, 379.00)	0.038^*^
IL-22	36.67 (2.05, 86.54)	11.20 (1.42, 91.01)	0.874	Myo	125.00 (21.89, 583.83)	166.40 (23.72, 1247.00)	0.245
IL-33	8.76 (7.51, 8.76)	8.76 (8.76, 10.11)	0.161	HsCRP	10.00 (2.58, 23.28)	27.60 (6.80, 55.14)	0.004^*^
TNF-α	41.15 (21.84, 71.55)	45.50 (17.29, 89.73)	0.525	PCT	0.09 (0.04, 4.10)	0.48 (0.04, 7.42)	0.362
IFN-α2	6.46 (4.00, 20.38)	6.46 (4.00, 33.26)	0.92				

*Data are presented as n (%) for categorical variables and as median (upper and lower quartile) for continuous variables*.

* *P < 0.05*.

We identified significant correlations between each pair for all cytokines except IL-33 and IFN-β. In addition, IL-6, IL-10, and IFN-β were closely associated with certain laboratory indicators of hepatobiliary function. Similarly, hematocrit (HCT), tBIL, direct bilirubin (DBIL), indirect bilirubin (IBIL), TP, creatine kinase (CK), and myoglobin (Myo) were significantly associated with most cytokines except IL-33, which was not correlated with any of the indices.

### The Selection of Predictors and Model Construction

**Figure 2:**
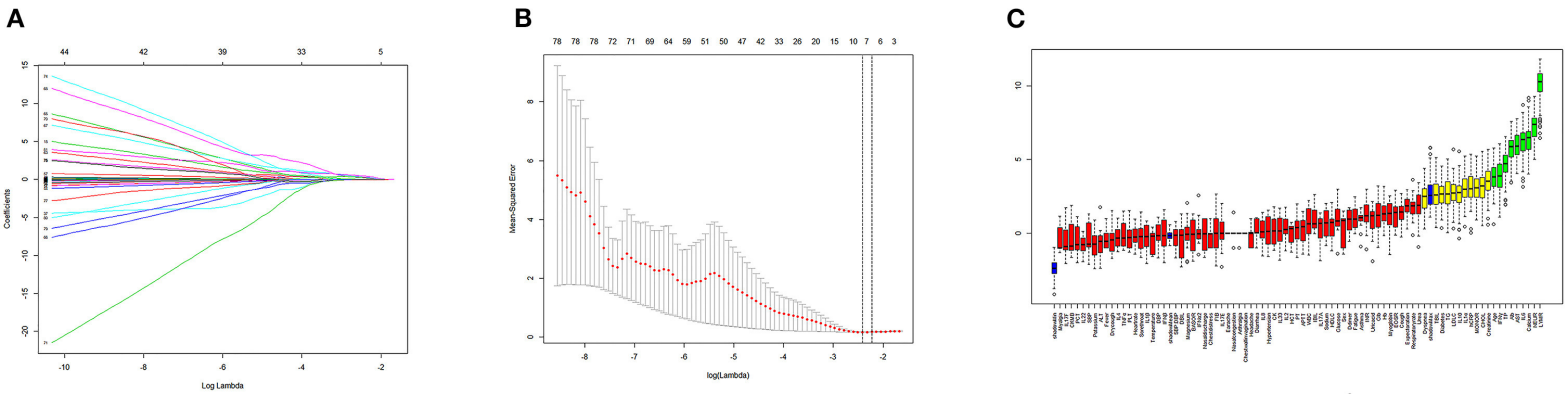
**(A)** Coefficient diagram of least absolute shrinkage and selection operator (LASSO) variables. Each curve in the figure represents the trajectory of the coefficient of an independent variable. The ordinate represents the value of the coefficient. The lower abscissa, λ, is a parameter that controls the severity of the penalty. The upper abscissa represents the number of non-zero coefficients in the model under the penalty parameter. **(B)** Adjustment parameters in the LASSO model; λ was screened by 10-fold cross-validation. A dashed vertical line was drawn at one standard error (1–SE standard) of the minimum and minimum standards. Λ 0.1 se corresponds to a model with good performance but the fewest number of arguments. **(C)** A variable importance plot according to Boruta feature selection. Blue boxplots correspond to minimal, average, and maximum Z scores of a shadow attribute. The Z-score clearly separates important and non-important attributes. Red, yellow, and green colors represent rejected, suggestive, and confirmed attributes by Boruta selection, respectively.

**Figure 3:**
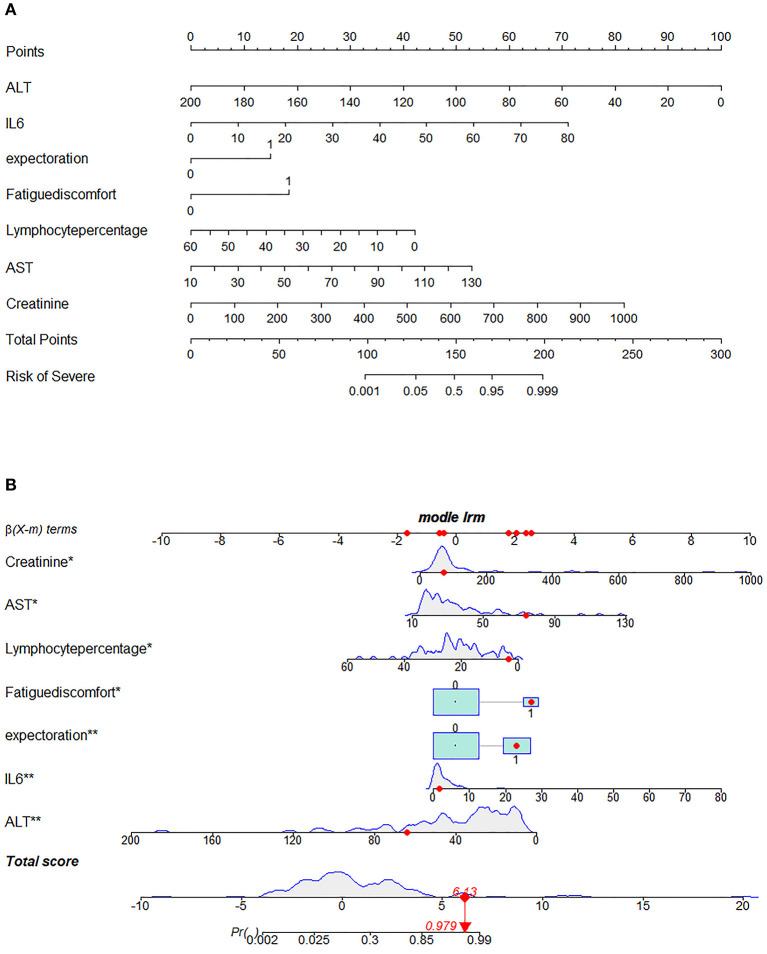
Nomogram for predicting severe coronavirus disease 2019 (COVID-19). **(A)** To use the nomogram for an individual patient, the points (top gridline) for each predictor variable are first assigned and the total points calculated. A vertical line from this value on the Total Points gridline then provides a probability for predicting severe COVID-19. The results of the binary variable are encoded as 0 and 1, representing the absence and presence of this symptom, respectively. The calculation is further illustrated in **(B)**, which shows the results of a patient with certain laboratory findings; the probability of this patient progressing to severe COVID-19 is 97.9%.

Next, we used variation analysis, LASSO regression, and the Boruta algorithm, to select three predictive panels and construct corresponding predictive models (predictive models A, B, and C, respectively) (Table 2, Figure 2). Predictive model B exhibited a better performance than the other two models in terms of sensitivity, specificity, discrimination, calibration, and clinical net benefit. In addition, the predictors included in this model are objective and universal. An optimal model, with seven features, alanine aminotransferase (ALT), IL-6, expectoration, fatigue, LYMR, AST, and serum creatinine (CREA), were used to generate a nomogram (Figure 3) and were encapsulated as an open-source online predictive calculator with R/Shiny (https://severeconid-19predction.shinyapps.io/SHINY/).

**Table 2:** Comprehensive performance of three prediction models.

	**Model A**	**Model B**	**Model C**
Variables	IL-6, LYMR, HsCRP, expectoration, dyspnea	ALT, IL-6, expectoration, fatigue, LYMR, AST, CREA	Dyspnea, diabetes, age, IFN-γ, IL-6, IL-10, LYMR, NEUR, AST, TP, Alb, Ca
AUC	0.8811	0.9104	0.8574
AIC	80.977	76.582	83.909
Cut-off	0.237	0.256	0.468
Specificity	0.831	0.842	0.958
Sensitivity	0.889	0.897	0.667
Hosmer-Lemeshow test (*P*-value)	0.1178	0.4989	0.2986

*AIC, Akaike's information criterion; AUC, area under the ROC curve*.

### Validation of the Online Predictive Model

**Figure 4:**
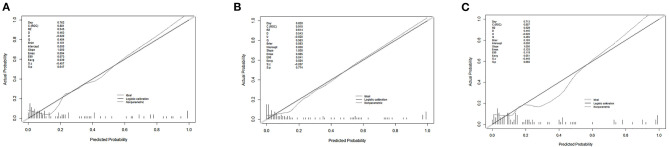
Calibration curves for the three predictive models. **(A)** Predictive model A, **(B)** predictive model B, and **(C)** predictive model C.

**Figure 5:**
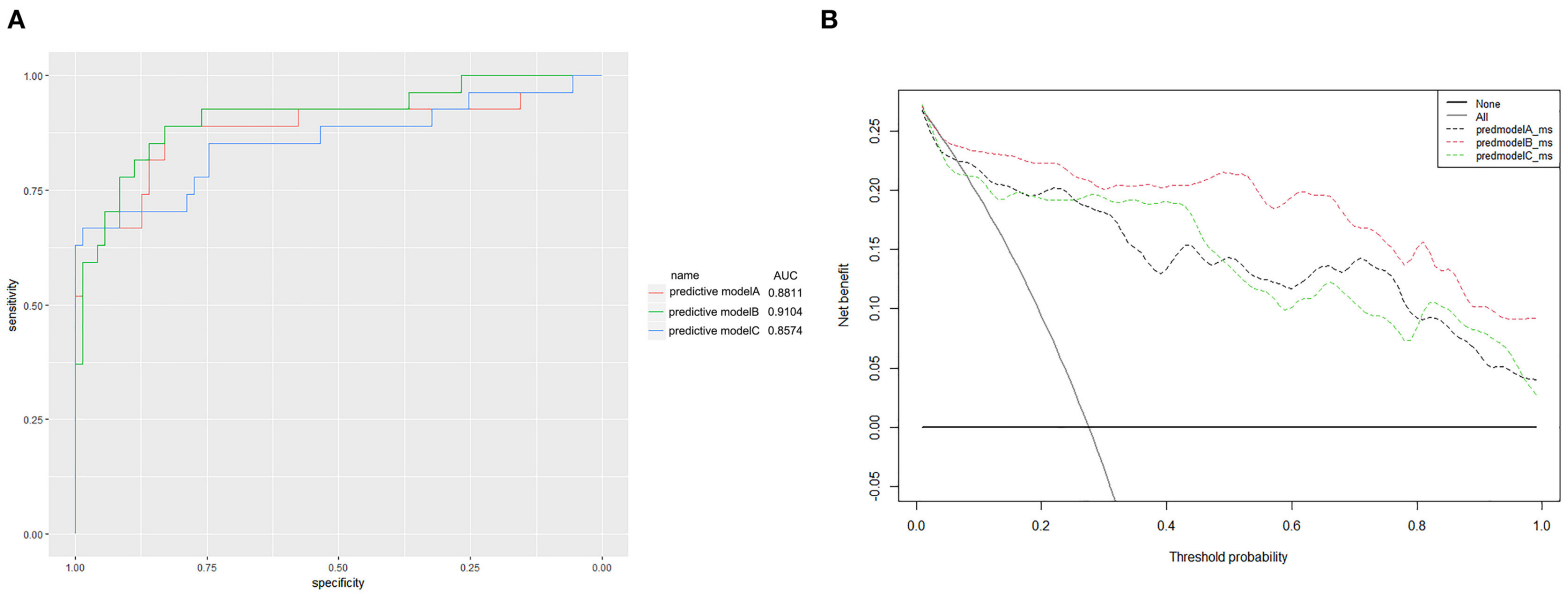
**(A)** Receiver operating characteristic curves for the three predictive models. The areas under the receiver operating characteristic curves were 0.8811, 0.9104, and 0.8574, for predictive models A, B, and C, respectively. **(B)** The decision curve analysis (DCA) of three predictive models.

Finally, we predicted the disease progression of the 108 patients in the validation cohort using our model. The model predicted that 18 patients would progress to severe COVID-19 while the remaining 90 would not. Compared with the follow-up results (91 patients with non-severe COVID-19 and 17 patients with severe COVID-19), the sensitivity, specificity, PPV, and NPV of our assay were 0.882 (95% CI; 0.622–0.979), 0.967 (95% CI; 0.890–0.991), 0.833 (95% CI; 0.747–0.896), and 0.978 (95% CI; 0.914–0.996), respectively. The model also achieved excellent discrimination (AUC = 0.879), calibration, and clinical net benefit (Figures 4, 5).

## Discussion

The accurate and individualized assessment of a patient who may progress to severe COVID-19 will promote the efficiency of clinical intervention and improve the rational use of medical resources. In the present study, we recruited 206 patients (162 patients with non-severe COVID-19 and 44 patients with severe COVID-19). We analyzed a range of indicators associated with severe COVID-19 and developed a novel predictive model that included ALT, IL-6, expectoration, fatigue, LYMR, AST, and CREA. This model proved to have excellent ability to predict the progression of COVID-19 during hospitalization, in both the derivation and validation cohorts.

Our final model was visualized in the form of a nomogram and was then packaged into an open-source and free predictive calculator (https://severeconid-19predction.shinyapps.io/SHINY/). The model represents a powerful tool with which to aid decision-making and guide treatment strategies for target patients who are at high risk of developing severe progression. The model could also be used to facilitate personalized management.

Previous research reported wide differences in the levels of a large number of cytokines from patients with non-severe and severe COVID-19 (26–28). Our present results identified obvious elevations of various cytokines in patients with severe COVID-19, including IL-1α, IL-1β, IFN-γ, TNF-α, IL-2, IL-4, IL-6, IL-10, and IL-17A. Of these cytokines, IL-6 and IL-10 showed the highest fold-change, thus indicating the presence of a strong inflammatory reaction; this could be a sufficient response to trigger a cytokine storm. Univariate logistic analysis showed that a number of cytokines can be used as predictors for patients with severe illness, although their predictive efficacies can vary considerably; these cytokines could not be used individually. We also found that underlying diseases (diabetes and hypertension), initial clinical characteristics (cough, expectoration, dyspnea, asthma, and debilitation), and laboratory findings [LYMR ALT, AST, CK, GlU, and procalcitonin (PCT)] were also significantly associated with disease progression, although these were non-specific. The extensive correlation between cytokines and the clinical response spectrum may be explained by multiple organ damage caused by the over-exuberant inflammatory response in severe COVID-19 (12, 29).

Univariate logistic analysis indicated that using a certain evaluation index could not provide sufficient evidence for the prediction of progression and that modeling by data mining may be a more efficient and viable tool with which to compensate for the lack of a single source of information (30). We used the LASSO algorithm and logistic regression and compared different modeling approaches. Finally, we selected a predictive model that included ALT, IL-6, expectoration, fatigue, LYMR, AST, and CREA. Our model achieved satisfactory predictive performance with AUCs of 0.910 and 0.879 in the derivation and validation cohorts, respectively. We also packaged this model into an open-source online format for clinical use. Although several predictive models have been published previously, these studies were associated with obvious limitations, including the fact that they were retrospective reviews or were associated with suboptimal predictive abilities or were not validated externally (31–33). Taking these limitations into account, our study is superior in several respects. First, we considered potential predictors for severe COVID-19 and included a comprehensive dataset retrospectively. Second, our shrinking model, featuring representative key variables, may exhibit better levels of performance than a complex model. This can be supported by the fact that our predictive model was established by comparing several different methods; the optimal method had a significantly higher AUC than the other models; this finding was reconfirmed in the validation cohort. Third, the predictive model was used to create a nomogram that was then used to generate an open-source online calculator format with visualization and maneuverability function.

There are also some limitations associated with our study that need to be considered. For example, we mainly focused on the changes of symptoms and the levels of key indicators in patients after SARS-CoV-2 infection and did not consider the influence of individual differences on the progression of disease. More in-depth investigations and longitudinal dynamic monitoring studies now need to be conducted to explain the specific characteristics of the potential predictors. Furthermore, the predictive model needs to be validated in a larger patient cohort and other populations outside of China.

## Conclusion

In this study, we developed and validated an online predictive calculator that provides personalized probability for the progression of disease based on seven commonly used variables. The model will be vital for early personalized management, to promote the appropriate allocation of medical resources, and to ensure that patients who may develop severe COVID-19 can receive appropriate treatment as soon as possible.

## Data Availability Statement

The original contributions presented in the study are included in the article/Supplementary Material, further inquiries can be directed to the corresponding author/s.

## Ethics Statement

The studies involving human participants were reviewed and approved by West China Hospital, Sichuan University Medical Ethics Committee. The patients/participants provided their written informed consent to participate in this study.

## Author Contributions

ZM and MW designed the research and wrote the manuscript. ZZ and YoZ responsible for the recruitment of COVID-19 patients and clinical treatment. YW and SG responsible for the detection of candidate biomarkers. ML, SY, and YaZ responsible for collecting and organizing data. All authors contributed to the article and approved the submitted version.

## Conflict of Interest

The authors declare that the research was conducted in the absence of any commercial or financial relationships that could be construed as a potential conflict of interest.

## Supplementary Material

Supplementary Figure 1 Box plots of continuous variables.

Supplementary Figure 2 Bar plots of categorical variables.

Supplementary Figure 3 ROC curves for each single cytokine and a combined panel of cytokines.

## Abbreviations

AIC: Akaike information criterion
ALB: albumin
ALT: alanine aminotransferase
APTT: activated partial thromboplastin time
AST: aspartate transaminase
AUC: area under the ROC curve
BASOR: basophil ratio
CHOL: cholesterol
CK: creatine kinase
CREA: serum creatinine
DBIL: direct bilirubin
DBP: diastolic blood pressure
DCA: decision curve analysis
DM: diabetes
EOSR: eosinophil ratio
FIB: fibrinogen
GLB: globulin
GLU: glucose
Hb: hemoglobin
HCT: hematocrit
HDL-C: high-density lipoprotein cholesterol
HLP: hyperlipidemia
HP: hypertension
HsCRP: high-sensitivity C reactive protein
IBIL: indirect bilirubin
IFN: interferon
IL: interleukins
INR: International Normalized Ratio
LDL-C: low-density lipoprotein cholesterol
LYMR: lymphocyte ratio
MONOR: monocyte ratio
Myo: myoglobin
NEUTR: neutrophil ratio
PCT: procalcitonin
PLT: platelets
PT: prothrombin time
ROC: receiver operating characteristic
SBP: systolic blood pressure
TBIL: total bilirubin
TG: triglyceride
TNF: tumor necrosis factor
TP: total protein
URIC: uric acid
WBC: white blood cell.
