# Supplementary figures and images for: Development and Validation of a Predictive Model for Severe COVID-19: A Case-Control Study in China
Source: Front Med (Lausanne). 2021 May 25;8:663145. doi: 10.3389/fmed.2021.663145 (PMC8185163; doi:10.3389/fmed.2021.663145)

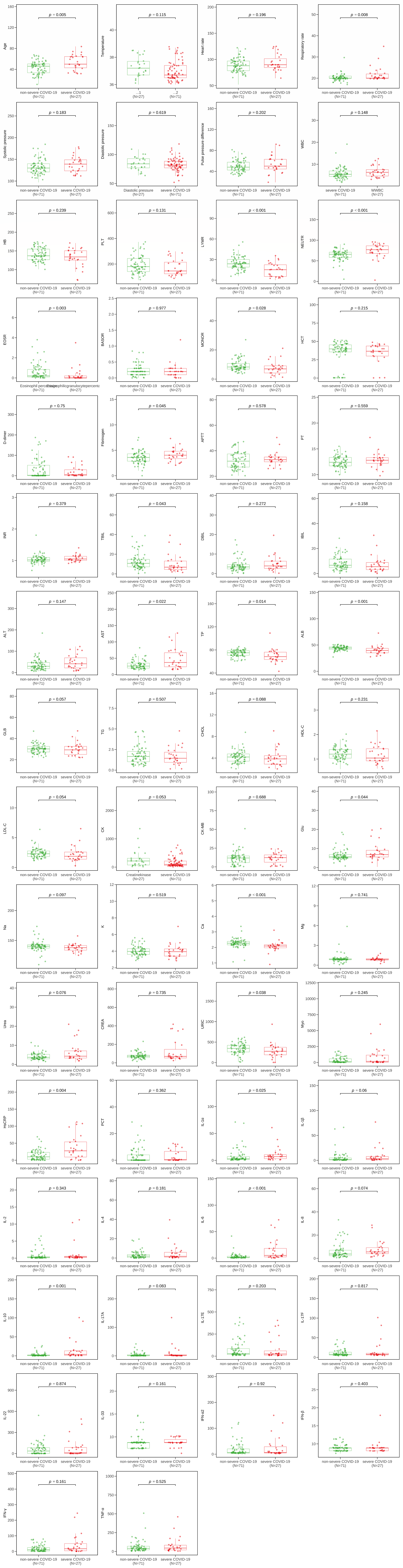

Supplement: Supplementary Figure 1 — Box plots of continuous variables. [file Image_1.TIFF]

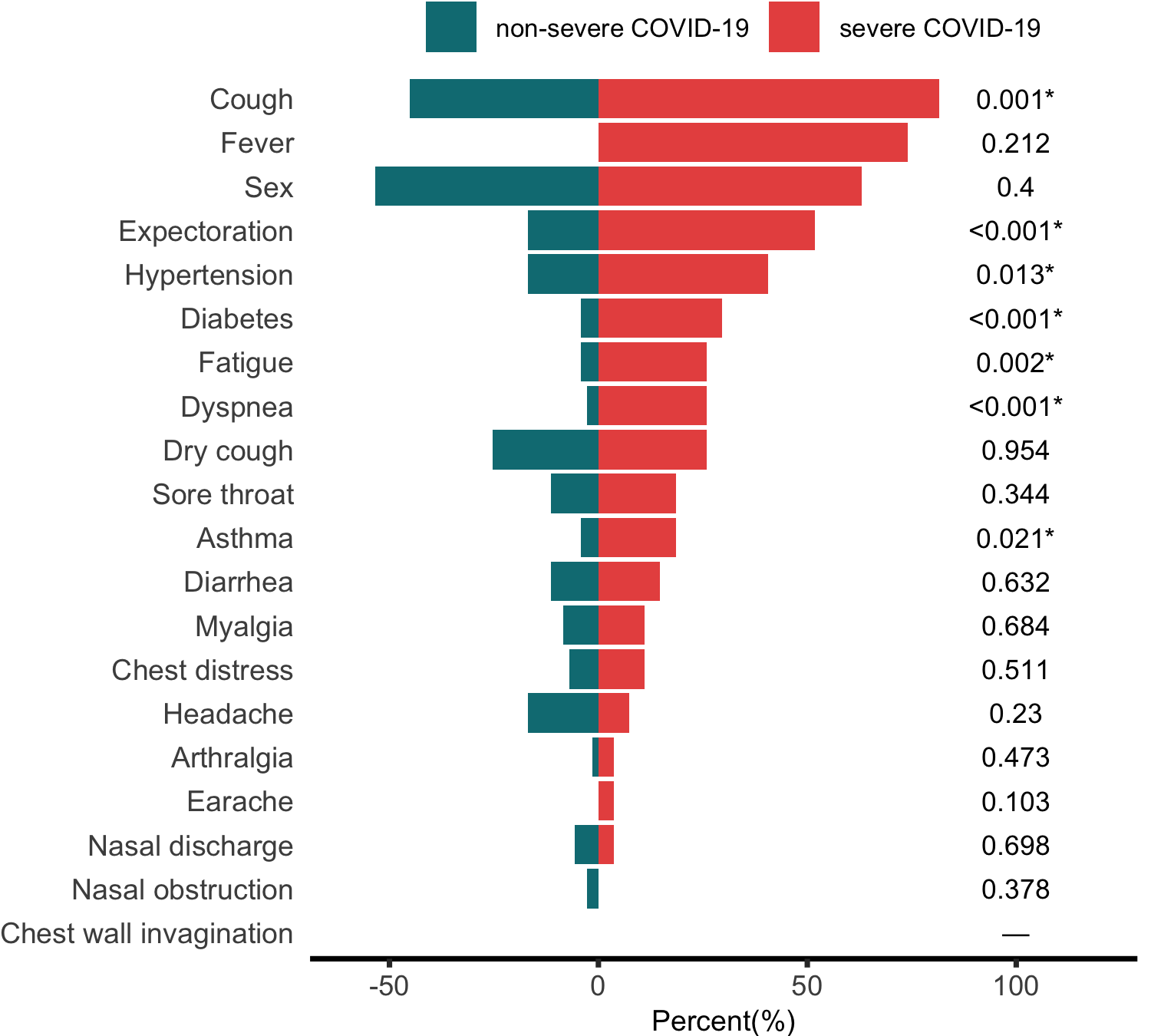

Supplement: Supplementary Figure 2 — Bar plots of categorical variables. [file Image_2.TIFF]

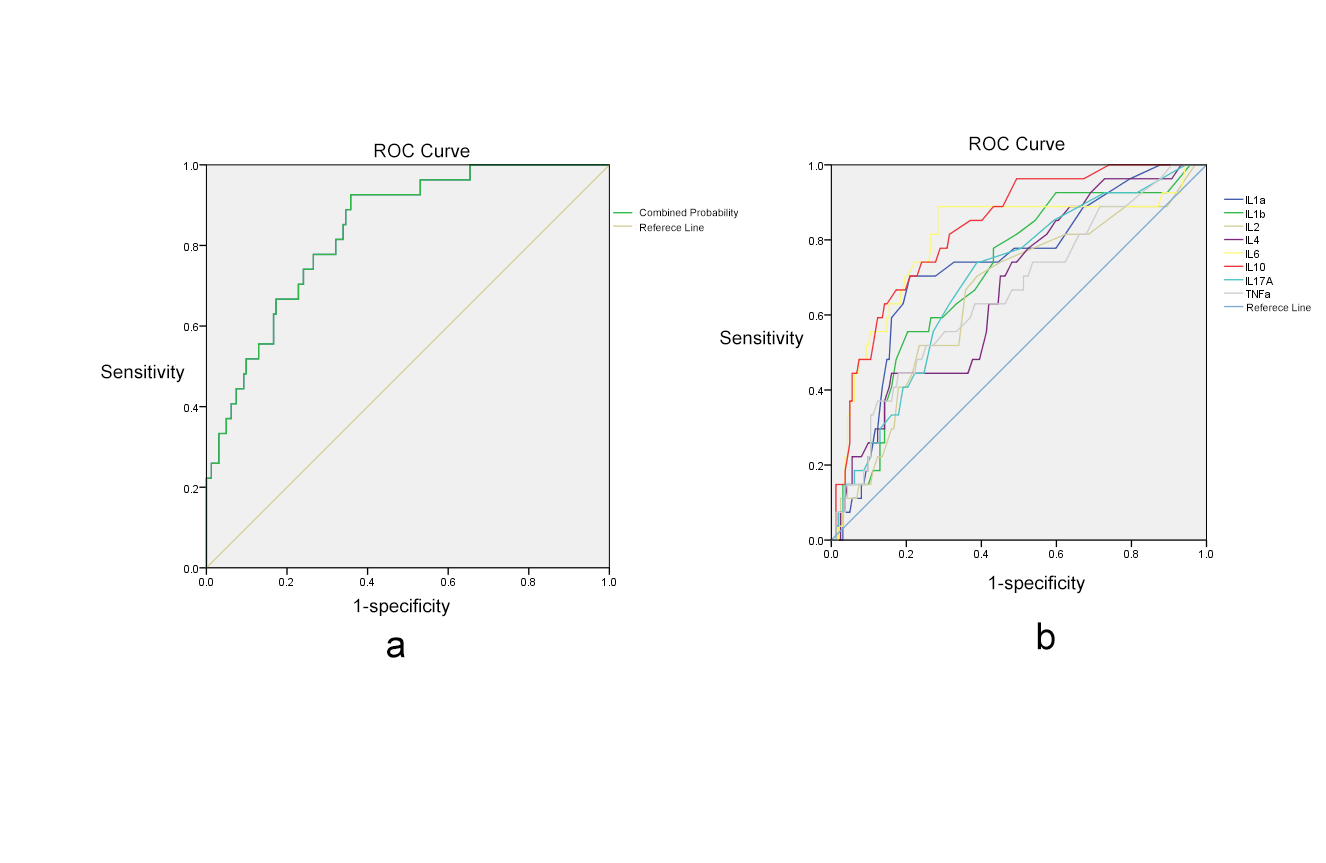

Supplement: Supplementary Figure 3 — ROC curves for each single cytokine and a combined panel of cytokines. [file Image_3.TIF]
